# Characterization of *Burkholderia pseudomallei* from spontaneous melioidosis in a Bornean orangutan

**DOI:** 10.14202/vetworld.2020.2459-2468

**Published:** 2020-11-18

**Authors:** Vincentius Arca Testamenti, Maryati Surya, Uus Saepuloh, Diah Iskandriati, Maryos Vigouri Tandang, Lia Kristina, Aris Tri Wahyudi, Dondin Sajuthi, Vivi Dwi Santi, Fiet Hayu Patispathika, Muhtadin Wahyu, Anton Nurcahyo, Joko Pamungkas

**Affiliations:** 1 Primatology Graduate Study Program, Graduate School of IPB University, Bogor 16128, Indonesia; 2 Primate Research Center, IPB University, Bogor 16128, Indonesia; 3 Borneo Orangutan Survival Foundation, Bogor 16128, Indonesia; 4 Department of Biology, Faculty of Mathematics and Natural Sciences, IPB University, Bogor 16680, Indonesia; 5 Department of Clinics, Reproduction, and Pathology, Faculty of Veterinary Medicine, IPB University, Bogor 16680, Indonesia; 6 Department of Animal Infectious Diseases and Veterinary Public Health, Faculty of Veterinary Medicine, IPB University, Bogor 16680, Indonesia

**Keywords:** *Burkholderia pseudomallei*, melioidosis, molecular characterization, nonhuman primate, orangutan

## Abstract

**Background and Aim:**

Melioidosis is a potentially fatal disease affecting humans and a wide range of animal species; it is often underdiagnosed and underreported in veterinary medicine in Indonesia. This study aimed to characterize morphological and molecular features of *Burkholderia pseudomallei*, the causative agent of melioidosis which caused the death of a Bornean orangutan.

**Materials and Methods:**

Pulmonary abscess samples were cultured on several types of media, including Ashdown agar, Ashdown broth, and MacConkey agar. Type three secretion system *orf* 2 real-time polymerase chain reaction (PCR) and latex agglutination tests were performed to identify the bacteria. Morphological characteristics were compared to all previously published morphotypes. Subsequently, the bacteria were characterized by multilocus sequence typing (MLST) and *Yersinia*-like flagellum/*Burkholderia thailandensis*-like flagellum and chemotaxis PCR. The results of the genotyping were afterward compared to all genotypes from Southeast Asia.

**Results:**

Multiple morphotypes of *B. pseudomallei* were perceived during the growth on Ashdown agar. Furthermore, it was identified by MLST that the Type I and Type II morphotypes observed in this study were clones of a single ST, ST54, which is predominantly found in humans and the environment in Malaysia and Thailand, although a very limited number of reports was published in association with animals. Moreover, the E-BURST analysis showed that the ST is grouped together with isolates from Southeast Asian countries, including Malaysia, Thailand, Singapore, and Cambodia. ST54 was predicted to be the founding genotype of several STs from those regions.

**Conclusion:**

*B. pseudomallei* ST54 that caused the death of a Bornean orangutan has a distant genetic relationship with other STs which were previously reported in Indonesia, implying a vast genetic diversity in Indonesia that has not been discovered yet.

## Introduction

*Burkholderia pseudomallei* is a saprophytic Gram-negative bacillus which causes melioidosis in humans as well as in a wide range of animal species. The causative bacteria can be found in water and soil in endemic areas [1]. It was predicted by a global epidemiological assessment that Southeast Asia, South Asia, Northern Australia, Western sub-Saharan Africa, and South America are included among areas with the highest risk for the occurrence of the disease [2]. Furthermore, an environmental suitability assessment suggested that melioidosis is probably endemic in 34 countries where cases have not yet been reported [2].

The considerably high morbidity and mortality rate of melioidosis is a major threat to the health of both humans and animals. An individual may acquire melioidosis through cutaneous inoculation, ingestion of contaminated water [3], or inhalation of aerosols and dust [4,5]. The fatality rate of melioidosis cases was 43% in Indonesia [6], 40% in Northeast Thailand, and even reached 90% in patients with severe sepsis [7]. In Indonesia, the case fatality rate was identified to be 43% [6]. Moreover, the causative agent was ­classified as Tier 1 Select Agent by the Centers for Disease Control and Prevention, USA due to its potential for bioterrorism [8], including its possibility to be transmitted through inhalation and ingestion routes, its high persistence in the environment, and the high prevalence of severe sepsis [9]. The first case associated with melioidosis in Indonesia was discovered in the beginning of the 20^th^ century, and throughout the decades, melioidosis was reported in several main islands of Indonesia, including Java, Sumatra, and Sulawesi [6]. However, many melioidosis cases went unreported, including 55 melioidosis cases from only a few hospitals in Indonesia [6]. Moreover, reports in association with veterinary melioidosis cases in Indonesia were also limited, and only included cases about three long-tailed macaques exported to the United Kingdom [10], one pig-tailed macaque exported to the United States [11,12], one cynomolgus macaque in a primate research center [13], and one Bornean orangutan in a rehabilitation center [14]. Interestingly, all the reported cases of melioidosis in veterinary medicine in Indonesia were observed in non-human primates (NHPs).

In Borneo Island, *B. pseudomallei* has been observed in humans, animals, as well as in other environmental samples (Table-1) [6,14-23]. The first reported melioidosis case in the Indonesian part of Borneo occurred in 2010 and since then melioidosis reports in both humans and animals have been continuously published from South Kalimantan, East Kalimantan, and various regions of the Malaysian Borneo.

**Table-1:** Reports on the infection or presence of *B. pseudomallei* in Borneo Island.*

References	Year	Source	Region
[15-20]	1964-2019	A total of 74 patients	Various regions in Malaysian Borneo: Kota Kinabalu, Sibu, Miri, Kapit, and Bintulu
[21,22]	1967-1976	Environmental samples in five regions	Various regions in Malaysian Borneo: Tawau, Lahad Datu, Kota Belud, Apin-apin Keningau Tenom, and Sandakan
[22]	1974	1 orangutan	Sandakan, Malaysia
Unpublished report listed in [6]	2010	1 patient	Banjarmasin, South Kalimantan, Indonesia
[23]	2016	13 patients	Samarinda, East Kalimantan, Indonesia
[14]	2017	1 orangutan	Samboja, East Kalimantan, Indonesia

* Data selected from http://melioidosis.info

In human medicine, melioidosis is often difficult to be diagnosed clinically due to its non-characteristic and multisystemic lesions, including respiratory infection [24], skin and soft tissue infection [25], genitourinary infection [26], bacteremia, neurologic lesions [27], as well as bone and joint disease [28]. The challenge in veterinary medicine is even greater since prominent clinical manifestations of melioidosis from one animal species to another may differ.

Culture in the laboratory is considered to be the gold standard regarding the diagnosis of melioidosis. *B. pseudomallei* is grown on routine media, such as blood agar and MacConkey agar (MCA), although the use of selective media, such as Ashdown agar and selective broth is highly recommended, which would significantly increase the yield of detection [29]. Ashdown agar is comprised of trypticase soy agar with a final concentration of 4% glycerol, 5 mg/L crystal violet, 50 mg/L neutral red, and 4 mg/mL gentamicin [30]. On Ashdown agar, single or multiple morphotypes of *B. pseudomallei* can be present [31]. The multi-morphotypic nature of the colonies may lead to the misidentification of *B. pseudomallei* colonies as contaminants [32]. Therefore, identification based on the morphological characteristics alone requires a proper level of expertise.

Considering the difficulty of diagnosing melioidosis based on morphology alone, a confirmation assay is needed for the identification of *B. pseudomallei*. The most specific confirmation assay ­available today is the real-time polymerase chain reaction (PCR) of type three secretion system (TTSS-1) developed by Novak *et al*. [33]. Besides, other diagnostic methods are also available, such as biochemical identification with either manual (API^®^ 20NE) or automated systems such as VITEK^®^ (bioMérieux, France) and BACTEC^®^ (Becton Dickinson, USA), as well as serological methods (latex agglutination test, IHA, and ELISA). However, the most commonly used method remains to be the real-time PCR of TTSS-1 due to its high specificity and sensitivity.

Another useful technique in the detection and grouping of *B. pseudomallei* strains is the *Yersinia*-like flagellum/*Burkholderia thailandensis*-like flagellum and chemotaxis (YLF/BTFC) PCR developed by Tuanyok *et al*. [34]. It is known that due to horizontal gene transfer, either the BTFC gene or the YLF gene is possessed by *B. pseudomallei exclusively*. The BTFC group is predominantly found in Australia, whereas the YLF group in Thailand and other SE Asian countries. Furthermore, the YLF group is commonly found in clinical samples, whereas the BTFC group is mainly identified in environmental samples [34].

After the confirmation of the diagnosis, genotyping is often performed for further characterization of the bacteria. The most widely used method for the *B. pseudomallei* genotyping is multilocus sequence typing (MLST), which was developed by Godoy *et al*. [35]. Seven loci of the housekeeping genes are targeted by *B. pseudomallei* MLST, namely, the *ace*, *gltB*, *gmhD*, *lepA*, *lipA*, *narK*, and *ndh*. All of these seven genes are located on chromosome 1 and are separated by at least 80 kb. Moreover, each gene contains several polymorphic sites, from which each unique strand is designated with an allele number. An ST number is designated on every possible unique combination of the seven alleles. In addition, the sequence, profile, and isolate data can be obtained from the *B. pseudomallei* MLST database, which is a free-access, online database available at http://pubmlst.org/bpseudomallei. At present, the database curates more than 6000 isolates from around the globe and more than 1700 STs are designated. Besides, the database also records data such as country of origin, source of the isolate, allele numbers, as well as travel history, and disease presentation.

The awareness of melioidosis among healthcare workers in Indonesia is low [6], and despite the great magnitude of burden, the disease is not categorized as a notifiable disease in Indonesia. However, efforts have been made to increase the awareness and diagnostic capacity, and to encourage the reporting of new cases. The complete epidemiological data on the disease would be beneficial for scientists during the evaluation of melioidosis cases, and it could also be used by health authorities to plan surveillance, mitigation, and prevention programs.

To the best of our knowledge, no bacterial morphology and molecular reports have been published for veterinary melioidosis cases for animals reared in Indonesia. At present, all the available molecular reports from Indonesia are about animals that were exported and diagnosed abroad. Moreover, the morphological and molecular characteristics of local *B. pseudomallei* would aid microbiologists in the more accurate identification of the bacteria. This study aimed to compare morphological and genotypic characteristics of the isolate found in this study to previously reported strains, in order to describe the morphological and genetic diversity of *B. pseudomallei* in Indonesia.

## Materials and Methods

### Ethical approval

Given the conservation status of the animal, the use of postmortem biopsy samples for diagnostic purposes was regulated and approved by the Nature Conservation Agency, Ministry of Environment and Forestry, Republic of Indonesia. An approval from the IACUC was not considered applicable due to the nature of this study that utilized postmortem samples with no involvement of live animals (as confirmed by consulting with the IACUC of Primate Research Center, IPB University).

### Animal

The study was performed on the postmortem samples of a 13-year-old, female Bornean orangutan (*Pongo pygmaeus wurmbii*) at the Central Kalimantan Orangutan Rehabilitation and Reintroduction Program in Nyaru Menteng (a Borneo Orangutan Survival Foundation/BOSF program), Indonesia in February 2019. The animal was examined after exhibiting signs of lethargy and tremor. During the physical examination, nasal discharge and a recovering wound on the leg were observed for the animal. Subsequent rapid tests for dengue fever showed antibodies against the dengue virus. The animal experienced pyrexia (40°C) and vomiting before the arrival of death on the following morning. Afterward, necropsy was performed on-site, and biopsy samples of pulmonary abscess, spleen abscess, and liver abscess were sent to the Microbiology and Immunology Laboratory, Primate Research Center of IPB University, for culture and bacterial identification.

### Bacterial culture

The pus sample was inoculated directly onto MCA and Ashdown agar (trypticase soy agar with 4% glycerol, 5 mg/L crystal violet, 50 mg/L neutral red, and 4 mg/l gentamycin). Plates were subsequently incubated aerobically at 37°C for 7 days. On day 2 and day 5, suspected *B. pseudomallei* colonies were subcultured onto new Ashdown agar, MCA, and tryptic soy agar (TSA) to obtain a pure culture. Afterward, bacterial growth and morphological characteristics were identified and recorded on a daily basis for 7 days. Colony morphotype was categorized according to the morphotype identification chart by Chantratita *et al*. [31], which included the assessment of surface texture at the center of the colony, the circumference of the colony, colony diameter, and color. In addition, an in-house latex agglutination test was also performed on suspected colonies, which was kindly provided by the Mahidol-Oxford Tropical Medicine Research Unit. The latex agglutination test was based on the monoclonal antibody clone 4B11 which recognized the capsular polysaccharide of *B. pseudomallei* [36-38].

### Molecular identification and characterization

The molecular identification and characterization were performed at the Biotechnology Laboratory, Primate Research Center of IPB University. Subsequently, the bacterial genomic DNA was extracted from a pure culture plate of *B. pseudomallei* according to the bacterial DNA extraction method by QIAamp^®^ DNA Mini Blood Kit (QIAGEN, Hilden, Germany). A 115 bp sequence of the DNA was amplified using the real-time PCR method developed by Novak *et al*. [33], which is associated with the type III secretion system of *B. pseudomallei*. The assay was performed in duplicates on colonies from both morphotypes. In addition, the melting curve analysis was performed to assess whether the assay has produced specific PCR products. 500 nM from both the forward and the reverse primer (forward: 5’-CGTCTCTATACTGTCGAGCAATCG-3’, reverse: 5’-CGTGCACACCGGTCA GTATC-3’), 1X SsoFast™ EvaGreen^®^ Supermix (Bio-Rad, California, USA), and 25 ng of DNA template were contained in 20 μL of TTSS-1 real-time PCR mixture. The initial denaturation of the cycles was set at 98°C for 2 min, followed by 40 cycles at 98°C for 5 s and 57°C for 10 s. real-time PCR was run in an iQ5™ real-time PCR machine (Bio-Rad, California, USA). The results of the amplification and melting curve analysis were interpreted in a iQ5™ Optical System and cycle threshold (CT) values were determined.

To confirm the geographic distribution classification of the bacteria, the bacterial DNA was also characterized by the PCR for BTFC and YLF developed by Tuanyok *et al*. [34], which was performed separately by applying conventional PCR. A total of 25 μL PCR reaction mix contained 500 nM from both the forward and the reverse primer (BTFC forward: GGCAGCGTCGAACTGTTCTAG, BTFC reverse: CGAATCAATTCGTTTCCCTTGT; YLF forward: TGCTCGGCTTCCAGATCAG; YLF reverse: CGGTCAGTTGCCCGCTATT), 1X of MyTaq™ HS Red Mix (Bioline, London, UK), and 80 ng of DNA template. The cycles were set at 95°C for 2 min, followed by 35 cycles at 95°C for 30 s, 60°C for 30 s, and 72°C for 40 s, as well as a final elongation set at 72°C for 7 min.

For further characterization of the bacteria, the MLST targeting seven housekeeping genes of *B. pseudomallei* was performed according to Godoy *et al*. [35], with modifications. Furthermore, a total of 50 μL of PCR mixture contained 500 nM from both the forward and the reverse primer, 1× of MyTaq™ HS Red Mix (Bioline, London, UK), and 1.6 ng of DNA template. The initial denaturation cycle was set at 95°C for 5 min, 35 amplification cycles of denaturation were set at 94°C for 30 s, the annealing temperature of the respective gene was set for 30 s, and the extension was set at 72°C for 40 s, followed by a final elongation at 72°C for 10 min. The annealing temperature was set at 55°C for the *ace*, *gmhD*, and *ndh*, 60°C for the *lepA* and *lipA*, and 63°C for the *gltB* and *narK*.

Agarose gel electrophoresis was used for the visualization of unpurified PCR products before their subsequent transfer to a commercial laboratory for DNA purification and DNA sequencing. Sequences were analyzed using BioEdit [39]. The ST found in this study was compared to other STs found in Indonesia as well as other countries in Southeast Asia. ST profiles and isolate data from neighboring ­countries were obtained from the MLST database (pubmlst.org/bpseudomallei). STs were analyzed with goeBURST^®^ [40].

## Results

### Morphological characteristics

**Figure-1:**
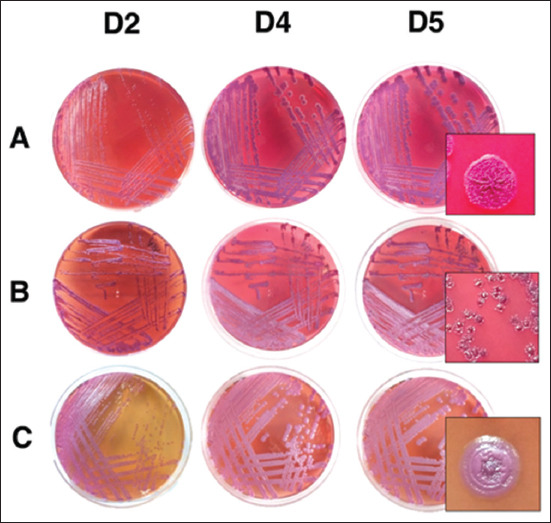
Morphology of *Burkholderia pseudomallei* colonies on Ashdown agar (row A and B) and MacConkey agar (row C) on day 2, day 4, and day 5. The mixed morphotypes on Ashdown agar were Type I (row A) and Type II (row B), which started to be distinctive on day 4.

Mixed *B. pseudomallei* morphotypes from the pulmonary abscess specimen were observed on Ashdown agar (Figure-1), whereas growth of *B. pseudomallei* was not observed from hepatic and splenic abscess specimens. A resemblance to the Type I and Type II morphology as described by Chantratita *et al*. [31] was observed for the colonies. The best observation of the colonies on Ashdown agar happened on day 4, as the morphological features, including the roughness and circumference of the colony became distinctive. On day 4, type I colonies exhibited features, including purple color, a diameter of more than 5 mm, and a wrinkled and rough surface texture with an irregular circumference. In contrast, type II colonies were perceived to be more translucent, pale purple in color, be about 2 mm in diameter, as well as having a smooth surface texture in the center with an irregular circumference (Figure-1). On MCA, the colonies were pale pink, umbonated, non-lactose-fermenting colonies with a metallic sheen. On TSA, the colonies were white and non-mucoid.

### TTSS-1 real-time PCR

**Figure-2:**
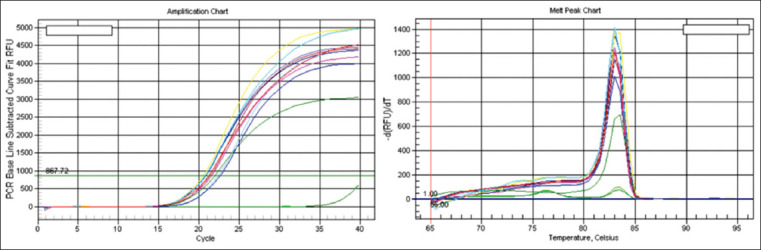
Amplification chart (a) and melt peak chart (b) of the type three secretion system quantitative polymerase chain reaction performed on *Burkholderia pseudomallei* colonies. The assay was run in duplicates for four colonies and a negative control. The mean cycle threshold was 21.44 and the melting temperature was set at 83°C.

A 115 bp region of the type III secretion system was amplified by the TTSS-1 real-time PCR assay. The average CT value was 21.44 for duplicates of multiple colonies. Melt curve analysis showed high specificity of the amplified region, and the melting temperature was at 83°C (Figure-2). Both morphotypes were confirmed to be colonies of *B. pseudomallei* by this essay.

### YLF/BTFC and MLST assays

The YLF/BTFC assay identified the presence of the YLF region and the absence of the BTFC region. The MLST PCR assay amplified all the seven target genes and both morphotypes were demonstrated to be identical by the sequencing results in the MLST alleles, with allele number 3, 1, 3, 3, 1, 2, and 1 for the *ace*, *gltB*, *gmhD*, *lepA*, *lipA*, *narK*, and *ndh* loci, respectively. Such a combination of loci is designated with ST54, a ST that was mostly reported to be found in both humans and the environment from regions in Thailand and Malaysia. Moreover, ST54 was also found in the UK, in people with a travel history to Asia. Before this study, there was only one report published on ST54 infection in animals, which occurred in a pig in Singapore [41].

### *B. pseudomallei* STs in Indonesia and neighboring countries

Most of the melioidosis case reports from Indonesia, considering either humans or animals, did not provide any information on molecular typing. The limited number of available molecular reports on *B. pseudomallei* in Indonesia is provided in Table-2 [10,11,42]. Only three STs (ST46, ST63, and ST54) have been reported, and they have a considerably distant genetic relationship, with only two or three loci matches among those STs.

**Table-2:** *B. pseudomallei* STs reported from Indonesia.

References	Year of the case	Source	Sequence type	Remarks
[10,42]	1992	Cynomolgus monkey	46	Animal from Indonesia, diagnosed in Great Britain
[10,42]	1992	Cynomolgus monkey	63	Animal from Indonesia, diagnosed in Great Britain
[10,42]	1992	Cynomolgus monkey	63	Animal from Indonesia, diagnosed in Great Britain
Currie*	2004	Human	46	N/A*
[11]	2012	Pig-tailed monkey	46	Animal from Indonesia, diagnosed in the USA
This report	2019	Orangutan	54	Animal from Borneo, Indonesia

* Details of the publication are not provided in the MLST database. *B. pseudomallei* STs. MLST=Multilocus sequence typing, *B. pseudomallei=Burkholderia pseudomallei*

**Figure-3:**
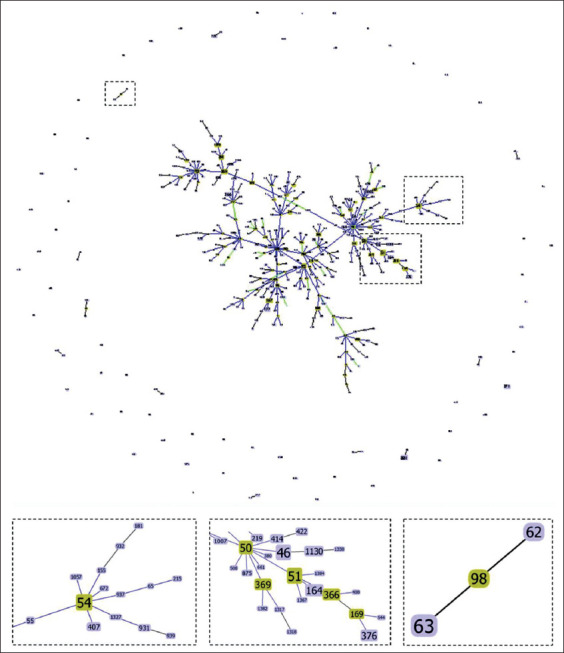
GoeBURST group snapshot of the sequence types found in Southeast Asia. Group definition was set at triple locus variants. The outlined sequence types are the predicted founding genotypes. In inset (a), ST54 was predicted to be the founding genotype of STs found in Southeast Asian countries, including Malaysia, Thailand, Singapore, and Cambodia. In inset (b), ST46 was shown to be closely related to the founding genotype ST50, which was mostly reported from South Asia. ST63 (inset c) was genetically distant from the large group of STs in SE Asia, with only one single locus variant and one double locus variant. ST=Sequence typing.

ST54 was previously reported in Malaysia, Thailand, Singapore, and the UK. ST54 was shown by the GoeBURST analysis to be clustered with several STs found in Asia (Figure-3). Furthermore, single locus variants of ST54, such as ST55, ST155, ST386, ST402, ST407, ST672, ST937, ST1057, ST1327, and ST1734, were found in Asian countries, including Thailand, Malaysia, Cambodia, China, Singapore, and Japan. ST46 can be considered as one of the most widespread STs in the world, which has been found so far in 13 countries of Asia, Australasia, and North America regions. Meanwhile, ST63 has only been reported in Indonesia.

## Discussion

Melioidosis cases in Indonesia have been reported since the 1920s [43]. However, the number of case reports is considerably low compared to other neighboring countries in Southeast Asia. The most recent review highlighted that there were altogether 101 published cases and at least another 45 unpublished cases of melioidosis in the country [6], which is due to that the national reporting system for the disease has not been established as melioidosis is not categorized as a notifiable disease in Indonesia.

All melioidosis reports in veterinary medicine in Indonesia were cases in association with NHPs. The outcome of all of those cases was fatal, including cases associated with three cynomolgus monkeys exported to Britain [10], one pig-tailed monkey exported to the US [11,12], one cynomolgus monkey in IPB Primate Research Center [13], and one orangutan from the East Kalimantan Orangutan Rehabilitation and Reintroduction Program in Samboja-Lestari (BOSF) [14]. Although four of the cases were diagnosed abroad [10-12], it is suspected that the animals were exposed to the causative bacteria in Indonesia.

Orangutans have been considered as the flagship species of NHPs and have gained global attention due to a significant decline in their number as well as the intense wildlife-human conflict. There are reports of melioidosis published on orangutans [14,22,44-49], all of which resulted in death. Borneo, as the main home for orangutans, has witnessed several fatal orangutan melioidosis in the territory, including the Sabah region in Malaysia [22,45], the Bandar Sri Begawan region in Brunei Darussalam [46], as well as the East Kalimantan [14] and Central Kalimantan Provinces of Indonesia. The *B. pseudomallei* infection may become one of the main challenges in the conservation of orangutans, considering that melioidosis cases have been reported from all around Borneo as well as the possibly-high susceptibility of orangutans to the disease.

To the best of our knowledge, there is very limited information available about the morphological and molecular characteristics of *B. pseudomallei* in Indonesia. In this study, the pathogen characteristics are outlined in comparison with previously reported isolates to support the regional epidemiology data that are available on melioidosis. Ashdown agar is a useful selective medium for the growth of *B. pseudomallei*. However, it still allows the growth of other bacteria, including multiple *Burkholderia* species (*B. thailandensis*, *Burkholderia multivorans*, *Burkholderia ubonensis*, and *Burkholderia humptydooensis*) as well as *Ralstonia*, *Cupriavidus*, and *Panoraea* [50]. The identification solely based on the ability of the species to grow on Ashdown agar and the colony morphology is not sufficient [50].

The latex agglutination test was performed as a rapid identification test. The kit used in this study had 98.7% sensitivity on *B. pseudomallei* isolates and 97.2% specificity when tested on related *Burkholderia* species [51]. The diagnosis was subsequently confirmed by the TTSS-1 real-time PCR method and also supported by the YLF/BTFC assay. The TTSS-1 orf 2 real-time PCR, which targets a type III secretion system, is still considered to be the gold standard for the identification of *B. pseudomallei*. The gene cluster is not present in most members of the *B. pseudomallei* complex; *B. thailandensis*, *Burkholderia oklahomensis*, and *B. humptydooensis*. However, even though the gene cluster is also present in *Burkholderia mallei*, 100% specificity to *B. pseudomallei* was demonstrated by the developed TTSS-1 orf 2 real-time PCR method [33]. Moreover, *B. pseudomallei* may exclusively have either the YLF or BTFC gene cluster, which was found to be beneficial during the differentiation of the geographical distribution of *B. pseudomallei* isolates [34]. The YLF gene cluster was ­identified in the bacteria isolated from the orangutan in this study, which is commonly expressed by strains from Southeast Asia, but not from Australia [34].

In addition, the mixed morphotypes (Type I and II) observed from the same clinical specimen in this study were demonstrated to be single ST by MLST, indicating clonality. The demonstration of mixed morphotypes by a single strain of *B. pseudomallei* is ­common. The classical colony morphology of *B. pseudomallei* is known to be the type I morphotype, and it may change its morphotype into several morphotypes related to starvation stress, iron deficiency, incubation temperature, and the presence of antibiotics [31]. Type II colonies produce significantly more biofilm in comparison with Type I colonies [31]. In this study, efforts to subculture type II colonies to new Ashdown agar media resulted in the growth of type I colonies.

Morphotypic switching may also occur during an infection, and it has been associated with invasive bacterial strains [31]. A previous study conducted by Gierok *et al*. [52] suggested that during acute infection, various morphotypes tend to switch toward a single morphotype as well as synchronize their metabolism. *B. pseudomallei* colonies grown on Luria-Bertani (LB) agar [53], TSA, and blood agar [54] were also found to exhibit morphology variation. *B. pseudomallei* colony variation observed on LB agar has been also linked to the ability of bacterial colonization in the stomach [53]. Moreover, mucoid and non-mucoid types have been observed on TSA and blood agar, and it was proposed that the variation is caused by the antigenic variation of OPS [54].

MLST is the most common genotyping method for *B. pseudomallei*. Even though a study suggested no association between the STs and the outcome of the disease [55,56], it is a useful method for molecular epidemiology studies as it can cluster strains based on genetic relatedness and geographic distribution. Genotyping is also useful during the determination of the source of infection [57].

The *B. pseudomallei* isolate found in this report was identified as ST54, an ST that was first found in 1964 in Peninsular Malaysia. Most of the ST54 isolates in the MLST database were found in Peninsular Malaysia, although they were also discovered in Thailand, Singapore, and the United Kingdom. ST54 was mostly isolated from human or environmental sources. Furthermore, there was only one report published previously on ST54 infection in animals, which occurred in a pig in Singapore [41]. The uncommon cases in the UK were discovered in patients with a travel history to Asia (http://www.pubmlst.org/b.pseudomallei).

E-BURST analysis of the MLST dataset has been proven to be a useful tool for describing the genetic relatedness of *B. pseudomallei* [35]. By connecting STs based on their relatedness in allelic profiles, the founding genotype of a group could be predicted by the E-BURST analysis, and thereby molecular epidemiology snapshots could be provided. The single-locus variants of ST54 include ST55, ST155, ST386, ST402, ST407, ST672, ST937, ST1057, ST1327, and ST1734. They were all identified in Asian countries, including Thailand, Malaysia, Cambodia, China, Singapore, and Japan. More specifically, ST54 was predicted to be the founding genotype of those STs.

To the best of our knowledge, there is no published report available on a successful treatment of melioidosis in NHPs. Infection caused by *B. pseudomallei* may be difficult to be treated since resistance to many antibiotics has been perceived for these bacteria [18,58-60]. In human patients, antibiotic treatment for melioidosis is divided into two phases, namely, Phase 1 for the acute infection and Phase 2 for the eradication [61]. The agents that are used during phase 1 treatment are usually cephalosporin (ceftazidime in most cases) and carbapenem (meropenem in most cases), whereas amoxicillin/clavulanic acid or cotrimoxazole is usually used during phase 2 [61].

The proper follow-up action of this report would be an environmental assessment in areas where orangutans are the most possibly exposed to the bacteria, including their natural habitat, rehabilitation facilities, and quarantine centers. The environmental assessment of *B. pseudomallei* may be challenging in areas where sampling has not been performed before. The sampling site should target a specific area that has a higher probability of the occurrence for the disease. So far, the environmental assessment has been conducted in the orangutan rehabilitation center on a small scale and needs to be done more extensively regarding the number of samples and the covered area. *B. pseudomallei* was not shown during the initial assessment in the environmental samples, however, it must be noted that the study did not cover a substantially large area of the rehabilitation center. Precautions are still needed during the design of rehabilitation and release programs since melioidosis cases in orangutans have been reported in some regions of Borneo Island. Moreover, precautions also need to be taken by people working in the forest or rehabilitation areas in Borneo, especially during the rainy season that is a period associated with a higher level of melioidosis incidence [62]. In the future, the genotyping of environmental and clinical *B. pseudomallei* isolates may provide a better picture of the distribution and transmission of the pathogen.

## Conclusion

*B. pseudomallei* was found to be the primary pathogen responsible for the death of a Bornean orangutan. The Type I and Type II morphotypes of *B. pseudomallei* on Ashdown agar were observed, but the MLST results indicated the clonality of the two morphotypes. ST54, the ST that infected this orangutan, is grouped with other STs found in Malaysia, Thailand, Singapore, and Cambodia, indicating the sharing of the ancestral genotype among STs in those regions.

## Supplementary Materials

The data supporting the findings of this study are openly available in PubMLST at http://pubmlst.org/bpseudomallei.

## Authors’ Contributions

JP, DI, and AN designed the study. MVT, LK, VDS, FHP, and MW performed the necropsy and tissue sampling. VAT, MS, and US conducted the laboratory work and analyses. VAT prepared the original manuscript. JP, DI, ATW, DS, and AN supervised the project. All authors read and approved the final manuscript.
